# The role of jealousy and infidelity in intimate partner violence against women: a qualitative meta-synthesis of five studies

**DOI:** 10.1186/s12889-025-24743-4

**Published:** 2025-10-15

**Authors:** Marjorie Pichon, Erin Stern, Vandana Sharma, Nambusi Kyegombe, Heidi Stöckl, Ana Maria Buller

**Affiliations:** 1 Gender Violence and Health Centre, Department of Global Health and Development, London School of Hygiene and Tropical Medicine, London, UK; 2 Harvard T.H. Chan School of Public Health, Boston, USA; 3 MRC/UVRI and LSHTM Uganda Research Unit, Entebbe, Uganda; 4 Institute for Medical Information Processing, Faculty of Medicine, Ludwig- Maximilians-Universität München, München, Germany; 5 Pettenkofer School of Public Health, München, Germany

**Keywords:** Intimate partner violence, Domestic violence, Family violence, Spousal abuse, Gender-based violence, Jealousy, Infidelity, Unfaithfulness, Meta-synthesis, Meta-ethnography

## Abstract

**Background:**

Jealousy and infidelity are frequently identified as key drivers of intimate partner violence (IPV) yet remain underexplored in IPV prevention programming and inadequately conceptualised in measurement frameworks. This study presents the first known meta-synthesis to compare findings across contexts, enhance interpretation, and generate transferable mid-range theories that elucidate the role of jealousy and infidelity in IPV.

**Methods:**

Using a meta-ethnographic approach, we synthesised findings from five purposively sampled qualitative studies produced by a joint Collaboration of authors, one in Ecuador (*n* = 100) and four in African countries: Ethiopia (*n* = 30), Rwanda (*n* = 224), Tanzania (*n* = 48) and Uganda (*n* = 40). Across all studies, women and men in heterosexual intimate relationships, aged 16–70 years were included.

**Results:**

The analysis identified 46 second-order and five third-order constructs linking jealousy and infidelity to physical, sexual, economic and psychological IPV, including controlling behaviours. At the community level findings highlighted traditional gender norms and community gossip that could fuel jealousy as mechanisms of controlling women’s behaviour. At the relational level male jealousy was instrumentalised as a socially acceptable means of controlling women, such as feigning jealousy to coerce sex. In contrast, women’s expressions of jealousy were typically more constrained, and often expressed through subverting traditional roles (e.g. refusing sex), which could result in violent consequences. At the individual level jealousy and infidelity were perceived as resulting from failure to adhere to hegemonic gender roles, further exacerbating IPV risk.

**Conclusions:**

To be effective, IPV prevention programmes must support the dismantling of patriarchal hierarchies while simultaneously addressing backlash to shifts in traditional gender norms. Interventions should also target jealousy and suspicions of infidelity to foster safer and more equitable relationships. Addressing these community, relational and individual dimensions is essential for mitigating the complex dynamics of jealousy, infidelity and IPV.

**Supplementary Information:**

The online version contains supplementary material available at 10.1186/s12889-025-24743-4.

## Introduction

### Jealousy, infidelity and IPV

Romantic jealousy is a commonly cited risk factor of intimate partner violence (IPV) against women but has been neglected in many violence response and programming efforts (e.g. [1, 2]). This is partially due to a lack of conceptual clarity as to whether jealousy precedes violence, is a mechanism to enact violence or, is a form of violence in and of itself. Evidence for the first two were presented in our 2020 global systematic review on the topic, where we found that jealousy experienced by both men and women could lead to physical and psychological violence against women, and that jealousy could be used by men to coerce sex, thus serving as a mechanism to enact sexual violence [2]. We also found evidence that women sometimes experienced accusations of infidelity from their partners as a form of psychological violence, but it was not always clear whether these accusations were rooted in feelings of jealousy, or whether they were used by men as a tool to reinstate dominance and control over their partner [2]. Nonetheless, the reviewed evidence indicates that jealousy is associated with greater uncertainty about one’s relationship and suspicions (whether valid or not) of infidelity, and both can lead to different forms of IPV against women [2].

This conceptual entanglement of jealousy, infidelity and IPV is also evident in measurement, as studies assessing IPV often include measurements of jealousy as a form of violence. For instance, the Revised Conflict Tactic Scale (CTS2), one of the most commonly used tools to measure IPV, includes several questions related to jealousy and suspicions of infidelity to measure psychological violence, thereby equating the two (e.g. “*How often does your partner become jealous or possessive”* and *“You know you can count on your partner to remain faithful to you”)* [3]. Moreover, jealousy has also been associated with controlling behaviours (e.g. [4]), which can include monitoring a partner’s whereabouts and limiting their contact with others [5]. However, there is no standardised tool to measure romantic jealousy in research [2], which can likely be attributed to the lack of conceptual clarity on how jealousy relates to IPV.

Born from these critical gaps, the Collaboration on Infidelity, Romantic Jealousy and IPV^1^ (henceforth the Collaboration) based out of the London School of Hygiene & Tropical Medicine was formed. The Collaboration aims to inform research and programming by exploring these understudied and undertheorized relational drivers of IPV. Towards this aim, the Collaboration produced six publications. The first was the aforementioned systematic review that identified three overarching mechanisms from infidelity and jealousy to IPV against women [2]: Suspicions of infidelity being associated with threatened masculinities and violence; Accusations of infidelity being associated with threatened femininities and violence; and Beliefs about infidelity and sex being associated with patriarchal culture and sexual violence. Additionally, the review highlighted several gaps in the IPV, jealousy and infidelity literature, including the need for more data on men’s experiences of violence, a greater focus on economic IPV and additional research conducted outside of the United States. The five subsequent publications produced by the Collaboration began to fill these gaps [6–10].

### The historical context of jealousy, infidelity and violence

Coming from the Latin word *zelus*, meaning ‘passion’ and ‘honour’, experiences of jealousy can be traced throughout history. Jealousy is a major theme in the Bible, as well as in Shakespeare’s *Othello* in which it is described as ‘venom’ and ‘misery’. While jealousy is always described as a strong emotion, the feelings associated with it vary by cultural and historical context [11]. It is generally conceptualised as an amalgamation of many emotions, including anger, sadness, embarrassment, sorrow, discontent, humiliation, shame, frustration, grief, insecurity, helplessness and unluckiness [12]. Jealousy occurring in romantic relationships is defined as “*a complex set of thoughts*,* feelings and actions that follow a threat to self-esteem and/or threaten the existence or quality of the relationship”* [13]. This is differentiated from envy, which occurs when one wishes to obtain something someone has, rather than stemming from a fear of losing something (e.g. a relationship one already has) [12]. In addition to resulting from fear, negative feelings of jealousy can also arise from love, possessiveness, and anger or sorrow at a situation [12].

The perceptions of, and meanings attributed to jealousy are varied. Researchers in the field of Evolutionary Psychology have described jealousy as a positive aspect of a relationship as it serves to protect men’s paternal certainty, and thus his genetic lineage (e.g. [14, 15]). Jealousy has also often been described as a key component of romantic love. As far back as 354AD, Saint Augustine, an influencer of modern Philosophy and Christianity, was quoted as saying “He that is not jealous, is not in love.” Furthermore, using the terms love and jealousy interchangeably, 17th century French author La Rochefoucauld stated “If love (and therefore jealousy) is judged by its effects, it resembles hate more than friendship” [16]. Perceptions of jealousy have also been historically gendered; while jealousy was successfully used as a legal defence for many femicides throughout the 18th −20th centuries in Western societies [17, 18], when experienced by women, jealousy has been more often described as a ‘petty’ emotion [12].

In modern day scientific enquiry, jealousy experienced by adults has been associated with insecure attachment to their parents during childhood [19, 20]. In adulthood, higher levels of jealousy have been associated with greater uncertainty about a relationship [21, 22], and decreased relationship quality and satisfaction for both partners [20, 23]. Jealousy within intimate relationships has been categorised into three types: reactive jealousy, which is caused by a partner’s behaviours with another person; anxious jealousy, which is caused by the possibility of a partner being romantically involved with someone else; and preventive jealousy, in which one acts to prevent their partner from becoming romantically involved with someone else [24]. Buunk’s framework allows us to conceptualise jealousy as multidimensional and elucidate various pathways through which jealousy and real or perceived infidelity drive relationship conflict leading to IPV. Moreover, it highlights the limitations of jealousy measurements, which have primarily centred on reactive jealousy, and are heterogeneously and inconsistently used in IPV programming evaluations [2]. We explore all three categories of jealousy within this meta-synthesis.

### The current study

We approach this work from a constructivist epistemology and employ a feminist approach. In doing so we attempt to be reflexive about the language we use, keeping it as neutral as possible, while remaining truthful to participant’s experiences. This has at times been challenging as participant’s perceptions may be influenced by external factors including programming; HIV programming in particular has often promoted monogamous relationships and thus may compound the stigma associated with having multiple sexual partners. We also use the term “romantic” jealousy to distinguish it from jealousy occurring outside of a romantic relationship, but in using this term we do not intend to romanticise jealousy or frame it in a positive light. Lastly, we acknowledge that the words infidelity and unfaithfulness have moral connotations within patriarchal discourse, however, we use them within this paper due to the lack of suitable alternatives.

The aim of this study is to synthesise the knowledge that has arisen from the Collaboration by developing new mid-range theories that help explain the role of romantic jealousy and infidelity in IPV against women. We hope researchers and practitioners working in the field of violence against women will find our insights helpful in informing their work and contributing to the identified gap around conceptual clarity on jealousy within this field.

## Methods

We conducted a qualitative meta-synthesis of five empirical studies on jealousy, infidelity and IPV to uncover new interpretations of the original authors’ interpretations and produce insights “greater than the sum of its parts” [25, 26]. We used a meta-ethnographic approach (one of several meta-synthesis methods) allowing us to translate findings from studies across contexts while preserving meaning [25]. This was achieved by re-analysing findings from multiple studies, breaking them down into pieces and building them up again to form a new, wider interpretation [27] that goes “beyond and behind” the original studies [28].

To aid this process, meta-synthesis methodology distinguishes between first-, second- and third-order constructs [29, 30]. Schütz (1962) describes first-order constructs as “participant’s ‘common sense’ interpretations in their own words” [29]. First-order data includes verbatim quotes from participants reported in the studies included in a meta-synthesis. Second-order constructs are the interpretations and findings of the authors of the included studies, based on participant data (i.e. first-order constructs). The authors of a meta-synthesis further abstract these second-order constructs to develop third-order constructs, which are the meta-synthesis authors’ interpretations of the findings presented by authors of the included studies.

Using this approach, we developed transferable knowledge about the role of jealousy and infidelity in IPV against women [31, 32]. We conducted this meta-synthesis following six steps set forth by Noblit and Hare [33]: sampling; reading the studies; determining how the studies are related; translating the studies into one another; synthesising translation; and expressing the synthesis. Each step is further described below.

### Sampling

Traditionally the studies sampled for meta-syntheses have been determined through a literature review, however, over the past few decades, researchers have begun to employ purposive sampling approaches [31]. This is because of the vast increase in quantity of qualitative studies, and the need to limit the sample to allow overarching themes to emerge. Thus, the sample must be diverse, allowing for generalizability, while at the same time being homogenous enough to allow translation and synthesis between studies, and for thematic saturation to be achieved [31].

This study is a culmination of the work produced by the Collaboration, providing an opportunity to present our findings in one coherent piece. Thus, we purposively sampled the five published, peer-reviewed papers produced by the Collaboration for this meta-synthesis. We synthesised qualitative findings from the studies resulting from in-depth interviews (IDIs) and focus group discussions (FGDs) with participants; one conducted in Ecuador (IDIs = 48, FGDs = 8; *n* = 100), and four in African countries: Ethiopia (IDIs only; *n* = 30), Rwanda (IDIs = 54, FGDs = 24; *n* = 224), Tanzania (IDIs only; *n* = 48) and Uganda (IDIs only; *n* = 40). All included studies were secondary analyses of previously collected data exploring IPV against women. Across all studies, adult women and men between 16-70 years-old were included (Table 1).

**Table 1:** Details of included papers and participants

Authors (citation)	Country	Data collection method and sample size	Participant sex	Participant age range	Recruitment setting	Participant relationship status	Analysis approach	Year data collected
Abudulai et al. [6]	Ethiopia	30 IDIs	Women and men	17–70 years	Somali refugee community members, elders/religious leaders, health workers, UN/NGO workers, community-based organisation workers, policy makers and host community members of the Bokolmayo refugee camp.	Participants in monogamous and polygynous marriages, and unmarried participants	Exploratory deductive and inductive thematic analysis	2016
Aloyce, Mshana et al. [7]	Tanzania	18 IDIs	Women	27–57 years	Participants of the Maisha longitudinal cohort study in Mwanza.	Single, married, divorced and widowed participants	Deductive and inductive coding guided by the concept of hegemonic masculinity	2018
Aloyce, Stöckl et al. [9]	Tanzania	30 IDIs	Men	22–61 years	Densely and sparsely populated streets in two districts of Mwanza.	Married	Thematic coding using an iterative process and unanimous consensus	2019
Buller et al. [10]	Ecuador	48 IDIs + 8 FGDs with 52 participants. Total = 100	Women and men	16–66 years	Low-income households participating in a cash transfer and food assistance intervention in Sucumbíos and Carchi regions.	Married	Sequential, exploratory thematic analysis using a constant comparative method and data triangulation	2013
Kyegombe et al. [8]	Rwanda & Uganda	54 IDIs + 24 FGDs with 170 participants. Total = 224 in Rwanda; 40 IDIs in Uganda	Women and men	21–45 years in Rwanda; 25–47 years in Uganda	Participants of Indashyikirwa intervention in Rwanda, as well as opinion leaders, women’s safe space facilitators and community activists; Participants of SASA! intervention in Uganda.	In Rwanda, married and unmarried participants; In Uganda, although not all participants were formally married, their relationship conformed to how marriage was understood in the context.	Thematic analysis complemented by constant comparative and deviant case analysis methods	2014–2018 in Rwanda; 2012 in Uganda

### Reading the studies

We individually read and re-read all included studies, allowing ourselves to be “absorbed by the materials” [25]. We became immersed in each study, and intimately familiar with their findings. During this process we also individually began making notes of emerging themes that cut across multiple studies.

### Determining how the studies are related

We conducted our first workshop in April 2022 with all co-authors, during which we discussed the themes that arose while reading the studies. We explored how the studies were related to each other using three approaches: reciprocal translation analysis, in which we compared the studies and determined whether overarching concepts from one could be translated into others; refutational synthesis, which involved exploring contradictions between studies and deviant cases; and line-of-argument synthesis, where we began to develop a holistic picture of the role of jealousy and infidelity on IPV against women through combining the data [33, 34]. Through this process we created a list of the ‘key concepts’ that was adapted into a preliminary coding framework [35].

### Translating the studies into one another

We organised the ‘key concepts’ into themes that became overarching parent codes, such as “upstream sociocultural determinants linked to jealousy, infidelity and IPV”, and sub child codes, such as “hegemonic masculinities”. We double coded the included studies to test and refine the coding framework and to ensure consistency and reliability of coding, using NVivo 12 or Dedoose software, or coding manually. Our team consisted of authors of the included studies, and thus all data was double coded by researchers who did not partake in the original studies to mitigate the risk of bias. After individually coding the studies, the two researchers met to discuss issues that arose during coding, reconciling discrepancies and suggesting further refinements to the coding framework. These refinements primarily consisted of adding missing codes for themes that emerged from the data and had not originally been included in the coding framework, such as “women’s reactions to men’s jealousy.” We reconciled all coding decisions into a final coding framework using NVivo 12 software, and these codes became our second-order constructs. This process of translating the studies into one another is inherently interpretive, with themes arising inductively from the data [33]. We were careful, however, to preserve the meaning of the original studies by confirming all coding with the original study authors on the meta-synthesis team [36].

### Synthesising translation

We had a second workshop in July 2022 with five co-authors (MP, ES, VS, NK and AMB), during which we re-conceptualized the findings of the included studies to provide fresh explanations of the data. This was an analytical and creative process consisting of ‘bricolage’ [37], in which ideas, hunches and intuitive feelings of an individual were discussed and expanded upon by the rest of the team [25]. Thus, an atmosphere of openness and creativity was promoted, allowing ideas to flow freely and be built upon by one another. This process was also reflexive, as we aimed to mitigate subjectivity [38].

As the researchers shared ideas for third-order constructs, we took notes and discussed whether these could be applied to all the included studies. We developed a draft coding framework of these meta-themes and then coded all studies using them. Through this process we determined that some codes needed to be expanded to accommodate findings from all contexts. Themes were deemed to be third-order constructs when they appeared in the majority of studies, while those only found in one or two studies were reported as second-order constructs. During the coding process a new third-order construct arose from the text of all studies that had previously remained uncoded, “jealousy and infidelity as manifestations of perceived relational and structural failings of female gender roles.” The final coding framework consisted of our third-order constructs. These represent the mid-level theories that emerged from this research, and thus, the conclusions of the meta-synthesis on the role of jealousy and infidelity in IPV against women.

### Expressing the synthesis

We provides a table of our second-order constructs, a narrative description, and an example from the data in Supplemental Material 1. The table also lists which studies evidence for each second-order construct were found. If a study is not listed, it does not mean the theme does not apply in that context, but that we did not find evidence for it in the papers included in this meta-synthesis. Moreover, while this table includes all constructs that arose from the included studies, they do not include all themes present in each study, as those not directly related to the role of jealousy and infidelity in IPV against women were excluded. The findings and implications from this meta-synthesis are described in the results and discussion sections below.

## Results

In this section we describe the second- and third-order constructs elucidated from our analysis and include quotes as examples from the data.

### Second-order constructs

Across the included studies we identified 46 second-order constructs related to jealousy, infidelity and IPV against women: 23 in Ecuador, 21 in Ethiopia, 27 in Rwanda and Uganda, 26 in the study with Tanzanian men and 29 in the study with Tanzanian women. We describe each second-order construct and sub-theme(s) within it, for which there was evidence from at least two of the included papers from different countries (*n* = 34), to avoid giving weight to findings that emerged from only one study or a single country. In our narrative of results, we highlight trends across studies and explore contradictions between them.

#### Prevalence of jealousy

Participants perceived jealousy to be a very common experience across almost all contexts. Despite not being included in the topic guides of the included studies, it was a theme that arose repeatedly, especially when discussed in relation to risk factors for IPV.

#### Perceptions of jealousy

The data highlights that although jealousy was perceived negatively, it was also often seen as common and was normalised, allowing men to use it as a justification for violence. In Ecuador, for example, jealousy was perceived as a “*manifestation of love*” and “*desirable in a relationship*”, thus “*men used this acceptability often to exert control over their wife’s behaviours*” [10]. In Rwanda and Tanzania, while jealousy was sometimes perceived as a sign of love, it was more often perceived as harmful to relationships. In Rwanda, participants of the Indashyikirwa couples curriculum described learning about the consequences of jealousy including that it is a key risk factor for IPV, which may have partially explained this negative perception [8].

#### Perceptions of infidelity

We found that meanings community members ascribed to infidelity were often gendered, and the impacts of being unfaithful or suspecting one’s partner of infidelity differed for men and women. In the sub-Saharan African contexts, men being unfaithful was generally much more tolerated than women’s infidelity. There were some variations in the normative acceptance of male infidelity in Uganda, with some women being more accepting of infidelity as long as their husbands were discrete, and with other women expressing concern about acquiring sexually transmitted infections through their husbands’ sexual affairs [8]. In Ecuador, infidelity by either member of the couple was described as socially unacceptable, and as “*signifying the absence of love*” in a relationship [10].

Moreover, in Ecuador and Tanzania, a woman being unfaithful was seen as humiliating and emasculating for her partner. For example, the authors of the Tanzanian study described male participants viewing female infidelity as “*intolerable*” and “*an ultimate act of betrayal*” [9]. In Ecuador, women who were believed to have been unfaithful were also stigmatized, and rumours that she had a sexual affair were experienced as “*a direct threat to her femininity*” as she was not seen to be fulfilling societal expectations as a married woman [10].

#### Causes of jealousy experienced by men

Across all studies there was evidence of men experiencing jealousy when (A) a partner refused sex and (B) women were employed or gained status in the community. In all contexts except Ethiopia, there was also evidence of men experiencing jealousy because of a female partner (C) interacting with other men, and (D) being away from home, as well as (E) community gossip.

- A. The data suggests men experienced jealousy when their female partner refused sex because this “*implied that she was in a relationship with another man”* [6]; the implication being she had her sexual needs met elsewhere. In Tanzania, “*while not all men immediately associated their wives denying them sex with infidelity*,* they still viewed it as uncaring and disrespectful behavior”* [9].
- B, C, D. Men reportedly experienced jealousy when their wives joined the workforce because this often meant that women dedicated less time to domestic responsibilities, and men in Tanzania reported that this made them feel neglected [9]. In Ecuador and Uganda, men typically expressed jealousy because they feared their wives were interacting with other men at work [8, 10]. There was also strong evidence across most studies that men’s jealousy could be provoked by women interacting with other men outside of work contexts in the community [8–10].
- E. Community gossip often acted as a catalyst for this distrust and jealousy experienced by men. For example, in Tanzania gossip by neighbours or family members was “*often regarded as proof of betrayal”*, even when the rumours were not confirmed [9]; while in Rwanda there was evidence that community members sometimes “*intentionally destroyed”* families and relationships by spreading rumours about infidelity [8].

#### Men’s reactions to experiencing jealousy

There was strong evidence from all studies that men who experienced jealousy could react with physical, sexual, psychological and economic IPV, including controlling behaviours. There were many causes of jealousy experienced by men leading to different forms of IPV. For example, a common pathway to economic IPV and controlling behaviours was the fear that as women gained financial independence, they would gain the means to leave their partner, thus men restricted women from gaining employment, or if they did work, took control over how the money their wife earned was spent. The authors of the study conducted with women in Tanzania reported that due to jealousy men “*reduced household providing*,* restricting them [women] from working*,* stealing their money or refusing to pay loans they both agreed on borrowing from the women’s microfinance groups*” [7]. There was also evidence of men using accusations of infidelity when their partner refused sex to coerce sex. In general, *“men’s aggressive behaviors resulting from these feelings of jealousy either aimed at disciplining or punishing their female partners*,* and ultimately reinstating their dominance by forcing them to comply with gendered expectations dictated by traditional gender roles”* [9].

#### Causes of jealousy experienced by women

The authors reported women also experienced jealousy because of (A) community gossip, as well as because of their partner (B) interacting with other women and (C) decreasing their financial support.

- A. Rumours about a man’s infidelity were described as common by participants, and this reportedly led to much conflict between couples [8, 9]. The authors of the study in Rwanda and Uganda noted that rumours about a man’s infidelity were much less likely to lead to IPV than rumours about a woman’s infidelity [8].
- B, C. Women reportedly monitored and questioned their partners about their comings and goings, their phone calls and text messages, and how much food they ate (in Tanzania), to determine whether they had another sexual partner [6–8]. In polygynous relationships in Ethiopia there was also evidence of competition between co-wives for their husbands’ affection and financial resources [6]. The latter was also mentioned in non-polygynous unions in Tanzania and Uganda, where male financial support was essential to the family’s survival, and survival was threatened when limited resources were split with other women or families [7, 8].

#### Women’s reactions to experiencing jealousy

While women’s experiences of jealousy sometimes led to physical and psychological IPV against their male partners, more often quarrels arising from these conflicts led to IPV against women [6, 8]. Due to “*power asymmetries and structural constraints*” rooted in women being economically reliant on their male partners, women often could not act on their jealousy, resulting in “*solitude and anxiety for some”* [8].

#### Upstream individual determinants linked to jealousy, infidelity and intimate partner violence

Men’s harmful consumption of alcohol exacerbated the pathways from jealousy and infidelity to IPV [7, 10]. Men who consumed alcohol were more likely to cause their partners to experience jealousy by coming home late and were reportedly more likely to be unfaithful [7]. They were also more prone to believing gossip about their partners’ infidelity and reacting more swiftly to jealousy with physical violence when drinking [10].

#### Upstream sociocultural determinants linked to jealousy, infidelity and intimate partner violence

There was strong evidence of the impact of sociocultural determinants on these dynamics. For instance, across the studies, women’s economic dependence on men constrained women’s ability to express jealousy, thus impeding jealousy-related conflicts from arising [6, 8]. Hegemonic masculinities were also linked to men feeling entitled to control their partner, and to the belief that men are hypersexual, providing cultural legitimacy for men to have multiple sexual partners [6–10]. In contrast, femininities built on the belief that women should be sexually available to their husbands led to them being blamed for their husband’s infidelity [6, 8, 10]. Femininities were also built on women being submissive and faithful to their husbands, and if they were not, this could be a risk factor for men perpetrating IPV [9, 10].

#### Upstream structural determinants linked to jealousy, infidelity and intimate partner violence

Household poverty arose as the main structural determinant in these pathways [6, 7, 9]. Competing for resources made women concerned about their partner engaging in other sexual relationships [6, 7]. It could also contribute to women joining the workforce, which was closely linked to men experiencing jealousy and perpetrating IPV [6–10]. However, this relationship is nuanced, since women working can also reduce household poverty, and the need to ask their partner for resources, which can be a risk factor for IPV in resource constrained households.

#### Protective factors against intimate partner violence

In Ecuador, Ethiopia and Tanzania there was evidence of participants beginning to reject traditional, patriarchal gender roles closely linked to men experiencing jealousy and perpetrating IPV [6, 9, 10]. With women becoming more empowered through education and employment, they have more opportunity to leave violent relationships, although this also risks provoking men’s jealousy and subsequent IPV. Additionally, in all studies there was evidence that some participants rejected positive perceptions of jealousy as an attribute of love and recognised its harmful impacts on individuals and relationships [6–10].

### Third-order constructs

**Fig. 1:**
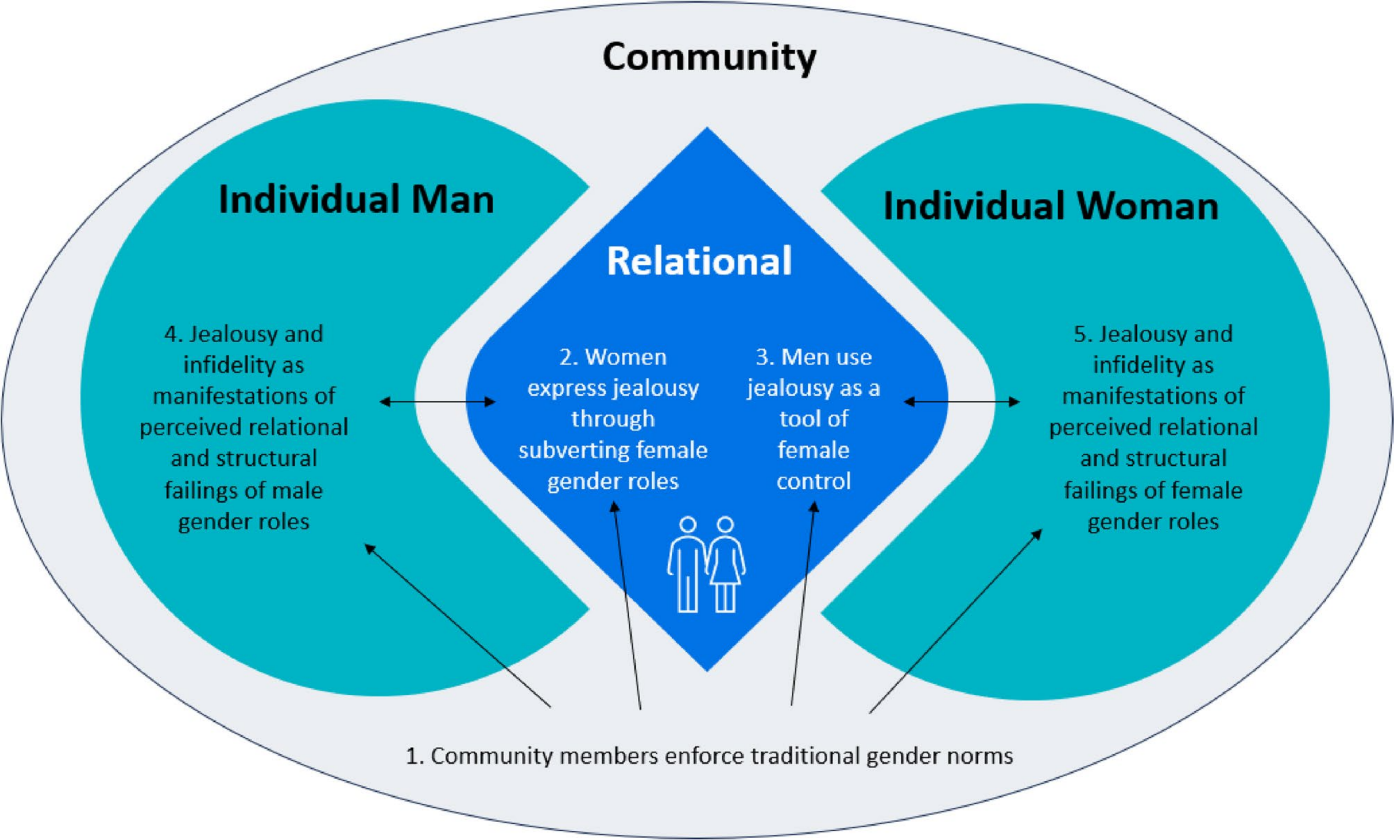
Third-order constructs relating to romantic jealousy and infidelity on the ecological framework conceptualising IPV [39]

These second-order constructs served as the “building blocks” for the five third-order constructs that arose from our interpretation [34]. The third-order constructs described below are the mid-range theories resulting from this meta-synthesis (Table 2) and serve as the basis for the recommendations arising from this work. Figure 1 provides a depiction of these third-order constructs mapped onto the ecological framework conceptualising IPV [39], highlighting at which levels interventions are needed to reduce IPV related to jealousy and infidelity.

**Table 2:** Third-order constructs relating to romantic jealousy, infidelity and IPV against women

Third-order constructs	Countries in which evidence was found	Studies in which evidence was found
1. Community members enfore traditional gender norms	Ecuador, Ethiopia, Rwanda & Uganda, Tanzania (men and women)	[6–10]
2. Women express jealousy through subverting female gender roles	Ecuador, Rwanda & Uganda, Tanzania ( women)	[6, 7, 9, 10]
3. Men use jealousy as a tool of female control	Ecuador, Ethiopia, Rwanda & Uganda, Tanzania (men)	[6–10]
4. Jealousy and infidelity as manifestations of perceived relational and structural failings of male gender roles	Ecuador, Ethiopia, Rwanda & Uganda, Tanzania (men and women)	[6, 8, 10]
5. Jealousy and infidelity as manifestations of perceived relational and structural failings of female gender roles	Ecuador, Ethiopia, Rwanda & Uganda, Tanzania (men and women)	[6, 7–9, 10]

We found that gender determines and influences all experiences of jealousy and infidelity; from causes of jealousy to reactions to infidelity, and who is blamed. For example, there was evidence from all studies of men using controlling behaviours and IPV when they experienced jealousy [6–10], while women were typically more constrained in how they could react to experiences of jealousy and were more likely to accept male infidelity as normal or inevitable [10]. Moreover, both men and women experiencing jealousy could often lead to men’s violence against women [7]. This gendered asymmetry was related to unequal power dynamics between women and men based on physical, social and economic positioning within society, among other factors.

#### Theory 1: Community members enforce traditional gender norms

Family and community members internalised traditional gender norms and used them to intercede in couple’s relationships and exert control over women’s behaviours. This is closely linked to community gossip, and more acceptance or tolerance of male infidelity compared to women’s infidelity, although male infidelity may still need to be hidden to not be shameful to men and/or their families.

#### Theory 2: Women express jealousy through subverting female roles

When women experience jealousy or suspect male infidelity, they may retaliate by not completing traditionally female roles (i.e. nurturing, being available for sex, or performing domestic duties at home). These behaviours that threaten attainment of hegemonic femininities, in turn can threaten their partners’ masculinities and can lead to IPV against women. Women’s behaviours to express jealousy also included confronting a partner about infidelity, flirting with other men, or perpetrating violence against men, although the later was rare.

#### Theory 3: Men using jealousy as a tool of female control

Our results suggest that it was common for men to use jealousy and related shame as a tool to control and coerce their partner. For example, feigning jealousy to coerce sex when they felt women had not fulfilled their gendered role of always being sexually available to them. This was linked to femininities in which women experienced accusations of infidelity as a form of psychological IPV and could feel compelled to comply with their husband’s requests for sex to avoid these accusations. Moreover, this construct is linked to the concept of jealousy being seen as a manifestation of love and thus being socially accepted as a tool of control.

#### Theory 4: Jealousy and infidelity as manifestations of perceived relational and structural failings of male gender roles

Gender norms are not static but are rather constantly changing and differ depending on setting. These changes provide opportunities to shift acceptance of jealousy, infidelity and IPV, but can also cause male backlash that risks increasing retaliatory IPV. Moreover, men who fail to meet social gender roles, such as providing financially for their partners, can feel their attainment of masculine ideals are threatened and may consume harmful levels of alcohol, have additional sexual partners, and suspect their partners of similar infidelity. In situations in which men fail to meet household needs, women may gain employment, which may cause male jealousy because her access to money is perceived to indicate she no longer needs him; or he may experience anticipatory jealousy and fear she will interact with other men and leave him for another man who can provide. This construct is also linked to female infidelity, which can be perceived as a failure on the part of men to “control” their partner, or to satisfy their partner sexually. Thus, men may use accusations of female infidelity as a way to coerce sex and reaffirm their perceived notions of masculinity within intimate relationships.

#### Theory 5: Jealousy and infidelity as manifestations of perceived relational and structural failings of female gender roles

When men accuse women of not meeting traditional gender roles, such as completing household tasks and being available for sex, they may threaten to or actually seek a new sexual partner. Some women, therefore, completed these tasks in anticipatory jealousy for fear their spouse had other partners or would find a new partner, including giving into sex to mitigate this risk. This construct was also sometimes linked to women flirting with other men and making their partner jealous or suspect infidelity, which goes against the expectation of women being sexually satisfied by one partner. Moreover, this construct can be linked to structural factors including female employment, which can increase women’s independence and threaten her intimate relationship. In turn, women’s empowerment can lead to anticipatory jealousy on behalf of men because they fear she will find a new partner. In challenging hegemonic femininities, these behaviours threatened their partner’s masculinities, thus sometimes leading to violence.

## Discussion

This qualitative meta-synthesis identified five mid-range theories elucidating the pathways from jealousy and infidelity to IPV against women. We mapped these theories onto the socio-ecological framework conceptualising IPV against women (Fig. 1). These findings align with our systematic review [2], which emphasized patriarchal gender roles and threatened masculinities and femininities as key mechanisms linking romantic jealousy with IPV. The current study builds on these insights, providing more detail into these mechanisms, and underscoring the gendered and relational dynamics fuelling IPV.

From a gendered lens, men were often afforded the social legitimacy to openly express jealousy, using it to assert control over their partners. Conversely, women were less able to explicitly express jealousy and instead conveyed it implicitly by subverting traditional female roles, such as withholding domestic chores or intimacy. Crucially, our findings also highlighted shifting gender norms across settings, which hold the potential to mitigate IPV by challenging patriarchal power structures. However, these changes can also provoke violent backlash when men seek to maintain control. These dynamics underscore the importance of addressing entrenched gender norms and relational power imbalances in efforts to prevent IPV.

### Programming recommendations

#### Targeting the community level

Our results indicated that community gossip - defined as casual reports about other people, typically involving details that are not confirmed to be true - played an important role in mediating jealousy between couples. For example, we found examples of men relying on neighbours as allies in controlling their partners movements, and as sources of evidence to ‘confirm’ their infidelity, leading to relational conflict and/or IPV. While third parties can cause relational harm, there is also evidence they can be leveraged to mediate conflict and promote peace and positive norms. For example, the *Bell Bajao* campaign in India encourages community members to ring a neighbour’s doorbell if they suspect violence [40]. This successful campaign increased bystander intervention, decreased community acceptance of IPV, and reduced stigma around experiencing violence, which promoted women’s help-seeking behaviours [40]. Thus, findings from the current study highlight the importance of working not only with couples, but also community members as they can play a major role in driving, or potentially mitigating violence and shifting broader norms related to such violence.

#### Targeting the relational level

Violence prevention programming could also help participants explicitly reflect on the ways jealousy and suspected (or actual) infidelity can be a risk factor for IPV and counter the harmful belief that jealousy is synonymous with love. For instance, the Indashyikirwa couples curriculum has a dedicated session on identifying the causes and consequences of jealousy and suspected (or actual) infidelity, highlighting how they are key IPV risk factors, and encouraging trust between couples through improved communication and honesty [8]. Towards the same goals, the SASA! and Indashyikirwa programmes also supported skill development such as communication and critical reflection to manage conflict, including around jealousy [8].

#### Targeting the individual level

Women’s economic empowerment interventions must carefully monitor and mitigate potential unintentional effects such as increasing IPV risk due to partner jealousy and suspicion of infidelity. This supports other research which has found that male partners may disapprove of or feel threatened by their spouse’s access to income, which can lead to backlash through physical, sexual, psychological or economic violence including controlling behaviours [41], particularly in settings where women’s economic participation is non-normative [42]. Our findings are in line with a recent review that identified reasons some men react negatively to women’s economic empowerment interventions, including feeling shame and loss of identity around being ‘replaced’ as the family’s primary financial provider, and fear that working will expose women to other romantic interests [43]. The literature also speaks to the importance of violence prevention programming shifting inequitable norms reinforcing men as the sole or primary financial provider, and emphasising the benefits of shared household roles [44].

Moreover, consistent with the existing literature [45], our results highlight the negative impacts that harmful alcohol use can have on relationships and the risk of IPV, by exacerbating the likelihood of experiencing jealousy or for men to react to suspected infidelity with violence. These findings align with a recent conceptual framework identifying the pathways between harmful alcohol use and IPV, which denotes how situational triggers including suspected or real infidelity, can lead to violence in the context of excessive drinking [46]. A systematic review examining the effectiveness of alcohol interventions combined with IPV programming found that while population- and community-level policies related to pricing, taxation, and regulations on the hours of alcohol sales and alcohol outlet density can be beneficial, the most effective programming worked with individuals [47]. Most of the alcohol and IPV prevention research, however, has been conducted in the United States, and more research in low- and middle- income countries is needed to determine whether these programmes can be effective across these different populations and contexts. Additionally, longer-term studies are needed to examine whether changes in alcohol and associated IPV are sustained.

### Recommendations for measurement

#### Quantitative

We recommend including measures of jealousy in IPV programming evaluations. Our findings support research linking jealousy with psychological IPV and controlling behaviours (e.g. [4]), but highlights the need to tease out emotional experiences of jealousy from these harmful behaviours in measurement tools, including the CTS2. The mid-range theories resulting from this meta-synthesis provide a good starting point to support enhanced measurement of jealousy in relation to IPV, as it highlights different experiences that may lead to jealousy and begins to disentangle jealousy from other related emotions such as fear or shame. As jealousy is a universal experience, the scale could be adapted for multiple contexts, but it is essential that translations are done carefully as words such as “unfaithful” are value-laden, and biased translations could distort the intended tone and meaning, affecting measurement validity and reliability.

#### Qualitative

More research is needed to determine the best way to ask about jealousy in qualitative research, so that questions are framed neutrally. As a starting point, we recommend asking about “sex outside of the relationship” rather than infidelity or unfaithfulness, which can promote stigma and have moral and religious connotations. Asking only about sexual activity, however, is insufficient as jealousy can be more subtle, arising from a suspected attraction. The tone with which questions are asked is also important; in the study conducted in Ethiopia participants were asked about their feelings towards their partners talking or spending time with other women. In doing so, however, the authors had to be careful not to perpetuate harmful attitudes towards co-wives or incite jealousy. Reflexivity is also essential when conducting jealousy research, and researchers must reflect on personal experiences, perceptions and feelings towards infidelity to mitigate potential bias [48].

### Recommendations for research topics

Firstly, future research on women’s reactions to experiencing jealousy are needed, as well as on comparing how jealousy and IPV manifest in different monogamous and non-monogamous relationships. Additionally, little is known about jealousy and IPV among displaced populations in humanitarian settings, and learning more is important as displacement contributes to changes in gender norms, marital and family structures and other jealousy-related risk factors for IPV, such as substance use and poverty [49, 50]. Given the strong gendered component of jealousy and IPV, additional investigation is also needed into how these dynamics manifest in queer and non-binary relationships and are related to IPV perpetrated against men.

Another area for future work could be on the link between anticipatory violence and reproductive coercion, as some men (or their families, or healthcare workers) may block their partner’s access to contraception for fear this would allow her to have sex with other people without consequences. Additionally, romantic jealousy may play a prominent role in driving the burgeoning rise of technology facilitated violence [51], particularly among young adults [52], and more research exploring the unique mechanisms and pathways from jealousy and infidelity to digital forms of IPV are needed. Finally, only one study included in this meta-synthesis explored jealousy from before relationships began, to after they ended. This temporal component is important as it is well established that violent incidents increase during or immediately after break-ups, and this is also when femicide is most likely to occur [53]. The literature is also sparse on the period of relationship formation, when feelings of possessiveness driving jealousy and jealousy-related violence typically begin.

### Strengths and limitations

We only sampled five studies produced by the Collaboration for this meta-synthesis, and thus our sample was relatively homogenous, and focused on jealousy, infidelity and IPV in cisgendered, heterosexual relationships in low- and middle-income countries. Moreover, only one included study was conducted outside of Africa, and only one was conducted with participants in polygynous relationships, which likely have different norms in that impact the pathways between jealousy, infidelity and IPV. Nonetheless, our meta-synthesis with this limited number of articles remains valuable, especially for exploring complex qualitative phenomena, and since the existing research is sparse. In addition, the perspectives of men were included in all studies, which is often lacking within the field. Moreover, all our third-order constructs, and all but two of our second-order constructs were supported by evidence from both women and men. While it was not possible to disentangle the perspectives of women and men in our synthesis, the inclusion of men’s perspectives in forming the conclusions of the original studies generates a more comprehensive and nuanced understanding of the dynamics at play regarding jealousy, infidelity and IPV against women.

There is a lack of consensus on whether to conduct quality assessments for a meta-synthesis [25]. Some researchers argue that quality appraisal improves synthesis rigour [54], however, this premise is not compatible with our social constructivist epistemology. Others point to the lack of valid and reliable quality criteria for qualitative studies [55], and thus subjectivity in assessing qualitative research quality [31]. The latter was of particular concern for this study, as the authors were also authors of the included papers. Authors remained reflective throughout the analysis and writing process to mitigate the risk of bias, and to ensure sole interpretation of second-order constructs in the included studies, rather than first-order constructs from the data. Including authors of the original studies also helped to maintain the meanings of the original studies, and appreciate the different contexts for participants [26]. This resulted in a rich and detailed synthesis of the evidence, offering important recommendations for future programming and research to prevent IPV.

## Conclusions

Jealousy and (suspicions of) infidelity are consistently identified as significant risk factors for IPV against women. This qualitative meta-synthesis enhances our understanding of the mechanisms and pathways linking these factors to IPV, offering improved conceptual clarity around the dynamics of jealousy and its association with violence. We identified five mid-range theories situated across the socio-ecological model, which provide actionable insights and highlight critical areas for future intervention efforts. Our findings underscore the pivotal role of gendered power hierarchies in shaping expressions of jealousy and its potential escalation to violence. These hierarchies influence who is socially “allowed” to experience and act on jealousy and perpetuate harmful dynamics within relationships. Addressing jealousy as a driver of IPV requires a dual focus: incorporating it in interventions to prevent its negative impact within relationships, while simultaneously challenging and dismantling the broader patriarchal norms and power imbalances underpinning these dynamics, to support safer and more gender equitable relationships and communities.

## Supplementary Information

Supplementary Material 1.

## Data Availability

No datasets were generated or analysed during the current study.

## Abbreviations

CTS2: Revised Conflict Tactic Scale
FGD: Focus group discussion
IDI: In–depth interview
IPV: Intimate partner violence
NGO: Non–governmental organization
UN: United Nations

## Footnotes

1
https://www.lshtm.ac.uk/research/centres-projects-groups/jealousy-ipv-collaboration.
