# Supplementary material for: The role of jealousy and infidelity in intimate partner violence against women: a qualitative meta-synthesis of five studies
Source: BMC Public Health. 2025 Oct 15;25:3502. doi: 10.1186/s12889-025-24743-4 (PMC12522318; doi:10.1186/s12889-025-24743-4)
Supplement: Supplementary file 1 — Supplementary Material 1. [file 12889_2025_24743_MOESM1_ESM.docx]

Supplemental Material 1. Second-order constructs and sub-themes relating to romantic jealousy, infidelity and IPV against women

| **Second order constructs and sub-themes** | **Narrative description** | **Example from text** | **Countries in which evidence was found** | **Studies in which evidence was found** |
| --- | --- | --- | --- | --- |
| Prevalence of jealousy | | | |  |
| Very common | Jealousy seen as a common emotion felt within the community, especially in connection to IPV. | *Male romantic jealousy was identified in almost all IDIs and FGDs as a major factor leading to intimate partner violence despite not being explicitly covered in the topic guides* (1). | Ecuador, Rwanda & Uganda, Tanzania (men and women) | (1-4) |
| Perceptions related to jealousy | | | |  |
| Normalisation | Jealousy is a normal, and sometimes positive emotion (i.e. demonstrative of love). | *Qualitatively, most participants referred to romantic jealousy as desirable in a relationship and thought that if a woman’s husband was not jealous he did not love her* (1)*.* | Ecuador, Rwanda & Uganda, Tanzania (women) | (1-3) |
| Negative | Negative views of jealousy, usually that it is an unhealthy and dangerous emotion. | *Although a few participants described romantic jealousy as a sign that her partner loved her or cared, the majority associated it with negative impacts on women in their intimate relationships* (3). | Ecuador, Ethiopia, Rwanda & Uganda, Tanzania (men and women) | (1-5) |
| Mutual in couples | When jealousy occurs in a relationship, it is usually both partners who are jealous. | *There was also consensus that if one member of a couple is jealous, it tends to result in mutual jealousy in a relationship* (2). | Rwanda & Uganda | (2) |
| Mentally unwell | Jealousy seen as an emotion experienced by people who are mentally unwell. | *Participants associated suspicions of female partner’s infidelity leading to IPV with their husband’s failure to regulate their emotions, calling this […] a medical disorder or obsession of men with their female partners* (3). | Tanzania (women) | (3) |
| Perceptions related to infidelity | | | |  |
| Normalization | Male infidelity is seen as normal, and something that their female partners tolerate. | *Despite the majority of participants claiming that infidelity by their female partners was an ultimate act of betrayal, they minimized their own infidelity acts and discussed them as common behavior that women needed to accept* (4). | Rwanda & Uganda, Tanzania (men and women) | (2-4) |
| Negative | Negative views of infidelity, usually that it is harmful to relationships and/or dangerous. | *Some participants described the infidelity in their relationship as a painful experience which fundamentally affected their wellbeing and increased their concerns about their health, particularly in relation to the risk of contracting HIV and other sexually transmitted infections* (2). | Ecuador, Rwanda & Uganda, Tanzania (men and women) | (1-4) |
| Perceptions related to IPV | | | |  |
| Negative | IPV seen as a negative behaviour. | *Participants reported that physically forcing one’s wife to have sex was considered machista and socially unacceptable* (1). | Ecuador | (1) |
| Sometimes justified | Although generally perceived negatively, IPV can be justified in certain situations including in response to infidelity. | *Most participants reported that physical violence was never justified in a relationship, but many made exceptions in cases of infidelity* (1). | Ecuador | (1) |
| Causes of jealousy experienced by men | | | |  |
| Female partner refusing sex | Men became jealous when their partner declined to have sex with them, often suspecting they were having sex with another man. | *[…] women refusing sex implied that she was in a relationship with another man* (5). | Ecuador, Ethiopia, Rwanda & Uganda, Tanzania (men and women) | (1-5) |
| Women in employment or gained social status | Men became jealous when their partners wanted to work outside the home, when they joined the workforce, and when they interacted with other men at work. | *When women did work, returning home later than expected or gossip […] about interactions with male colleagues could spark violence* (1). | Ecuador, Ethiopia, Rwanda & Uganda, Tanzania (men and women) | (1-5) |
| Female partner interacting with other men | Men became jealous when they suspected their partner of interacting with other men, including flirting to intentionally make them jealousy. | *Men were often described to be jealous or suspicious of women socialising with, or attracting the attention of, other men […]* (2). | Ecuador, Rwanda & Uganda, Tanzania (men) | (1, 2, 4) |
| Female partner away from home | Men became jealous when their partner left home or came home later than expected. | *[…] participants explained that they do not expect married women to come home late or leave home without their husband’s permission. Failure to abide to this standard suggested to the interviewed men that their partners were engaged with other men […]* (4). | Ecuador, Rwanda & Uganda, Tanzania (men and women) | (1-4) |
| Community gossip | Men became jealous after hearing gossip from community members about their partner’s behaviours. | *A few male participants also spoke about how families and relationships can be intentionally destroyed by others due to gossip and rumours around partner infidelity […]* (2). | Ecuador, Rwanda & Uganda, Tanzania (men and women) | (1-4) |
| Reduced attention from female partner | Men became jealous when their partner reduced the attention they showed them. | *It was reported that reduced love, care or attention from female partners was another action that led to romantic jealousy* (4). | Tanzania (men and women) | (3, 4) |
| Paternity doubts | Men became jealous when they doubted if they were the father of their partner’s child. | *Some men reported being provoked by a partner’s lover or realized that their children were the result of another partner* (4)*.* | Tanzania (men) | (4) |
| Female partner attempting to end relationship | Men became jealous after their partner suggested separation. | *Behaviours such as […] suggesting separation […] triggered emotions of romantic jealousy* (4). | Tanzania (men) | (4) |
| Women’s reactions to men’s jealousy | | | |  |
| Change own behaviours | Women changed their own behaviours to keep their partner’s from becoming jealous, including not refusing sex and not leaving home. | *Some women described adjusting their behaviours to protect against gossip. For example, one woman stopped leaving the house without her husband […]* (1). | Ecuador | (1) |
| Men’s reactions to experiencing jealousy | | | |  |
| IPV – Multiple forms | Men who experienced jealousy responded with multiple forms of violence against their partner. | *This manifested as physical, emotional, and economic IPV and controlling behaviours […] in response to anxious or preventive jealousy […]* (2). | Ecuador, Ethiopia, Rwanda & Uganda, Tanzania (men and women) | (1-5) |
| IPV – Physical | Men who experienced jealousy responded with physical violence against their partner. | *Thus, men were pressured to respond to gossip quickly to maintain their social status and this sometimes took the form of physical intimate partner violence, especially if they had been consuming alcohol* (1). | Ecuador, Ethiopia, Rwanda & Uganda, Tanzania (men and women) | (1-5) |
| IPV – Psychological | Men who experienced jealousy responded with psychological violence against their partner. | *[…] the majority associated it [romantic jealousy] with negative impacts on women in their intimate relationships. These included insults, accusations of infidelity, threats to separation […]* (3) | Ecuador, Ethiopia, Rwanda & Uganda, Tanzania (men and women) | (1-5) |
| IPV – Economic | Men who experienced jealousy responded with economic violence against their partner. | *Romantic jealousy was also the justification men gave for controlling their partners […] income, restricting them from working, making final decisions on their partner’s money […]* (4). | Ecuador, Ethiopia, Rwanda & Uganda, Tanzania (men and women) | (1-5) |
| IPV – Controlling behaviours | Men who experienced jealousy responded by controlling their partners behaviours. | *[…] in response to anxious or preventive jealousy […] men sought to exert control on women’s ability to work outside the home, leave the house, determine who they visited, as well as what they wore* (2). | Ecuador, Ethiopia, Rwanda & Uganda, Tanzania (men and women) | (1-5) |
| IPV – Sexual | Men who experienced jealousy responded with sexual violence against their partner. | *Sexual IPV because of romantic jealousy was less frequently described and captured situations of forced sex and excessive demand for sex by their male partners* (3). | Ecuador, Tanzania (men and women) | (1, 3, 4) |
| Non-partner violence | Men who experienced jealousy responded with violence against other men and self-harm. | *A considerable number of participants described violence towards their partner’s lover as a consequence of romantic jealousy, especially after proving their partner’s infidelity or being provoked by their partner’s lover* (4). | Tanzania (men) | (4) |
| Causes of jealousy experienced by women | | | |  |
| Community gossip | Women became jealous after hearing gossip from community members about their partner’s having another sexual partner. | *One male partner of a couple similarly described how someone had gossiped to his wife that he had had an affair, which was not true, and it created much conflict, which took significant efforts to resolve* (2). | Rwanda & Uganda, Tanzania (men) | (2, 4) |
| Partner interacting with other women | Women became jealous when they suspected their partner of interacting with other women. | *Women were more often described to question men about their whereabouts and intentions because of anxious jealousy or the suspicion of infidelity […]* (2). | Ethiopia, Rwanda & Uganda, Tanzania (women) | (2, 3, 5) |
| Reduced financial support | Women became jealous when their partners didn’t provide for them financially. | *Women treated their partners with silence or avoided confronting them after feeling jealous […] because their partners provided less for their family* (3). | Ethiopia, Rwanda & Uganda, Tanzania (women) | (2, 3, 5) |
| Women’s reactions to experiencing jealousy | | | |  |
| IPV - Physical | Women who experienced jealousy responded with physical violence against their partner. | *Several participants reported that women’s suspicions of her husband entering into a sexual relationship with other women could spark romantic jealousy, which could in turn lead to women being physically violent towards her husband* (5). | Ethiopia, Rwanda & Uganda | (2, 5) |
| IPV - Psychological | Women who experienced jealousy responded with psychological violence against their partner. They also experienced accusations of infidelity as a form of violence against themselves. | *Romantic jealousy experienced by women in polygynous relationships […] led to […] women using physical and psychological violence in retaliation for men marrying other women* (5). | Ethiopia, Rwanda & Uganda | (2, 5) |
| Confrontation | Women who experienced jealousy confronted their partner or the other woman about the infidelity. | *Confronting their partners following proven or suspected infidelity was one of the pathways that connected romantic jealousy experienced by women to IPV* (3). | Rwanda & Uganda, Tanzania (women) | (2, 3) |
| None | Women who experienced jealousy did not have the power to react. | *Women’s jealousy on the other hand is more often constrained by power asymmetries and structural constraints* (2). | Rwanda & Uganda, Tanzania (women) | (2, 3) |
| Change own behaviours | Women who experienced jealousy changed their own behaviours, including refusing sex, not cooking for their partner and focusing on work. | *In addition, participants narrated choosing to focus more on work as a way of coping with their partners once they became jealous or suspected infidelity* (3). | Tanzania (women) | (3) |
| Violence against non-partner | Women who experienced jealousy responded with violence against other women and children, and self-harm. | *Conflicts and physical violence between co-wives arising from romantic jealousy were also discussed by some of the participants* (5). | Ethiopia | (5) |
| Men’s reactions to women’s jealousy and retaliatory behaviours | | | |  |
| IPV – multiple forms | Men reacted to their partner’s being jealous, or their gendered retaliation, with multiple forms of IPV. | *For some of those who refused to have sex with their partners, they explained that this led to IPV, including insults, the accusation of having sex with another man, beating them, threats to leave them, and other vengeful acts that affected these women emotionally* (3). | Tanzania (women) | (3) |
| Upstream individual determinants linked to jealousy, infidelity and IPV | | | |  |
| Male alcohol consumption | Men drinking alcohol increased their feelings of jealousy, and they often committed IPV when they had been consuming alcohol. | *[…] alcohol consumption was described as an amplifier of men’s feelings of romantic jealousy and an important influence on men’s ability to emotionally control themselves* (3). | Ecuador, Tanzania (women) | (1, 3) |
| Upstream relational determinants linked to jealousy, infidelity and IPV | | | |  |
| Polygyny | Polygyny was linked to women experiencing jealousy leading to IPV. | *[…] in polygamous relationships, romantic jealousy experienced by women was elicited by an unequal distribution of financial resources and affection between co-wives […]* (5). | Ethiopia | (5) |
| Upstream sociocultural determinants linked to jealousy, infidelity and IPV | | | |  |
| Women’s economic dependence on men | Women’s economic dependence on their partner impeded them from leaving abusive relationships, and encouraged men to perpetrate economic IPV. | *These include their economic and social dependence on men for maintaining their social status and meeting their and their children’s financial needs. In turn this may have limited their ability to react to any jealousy that they may have felt and thus its contribution to relationship conflict and IPV* (2). | Ethiopia, Rwanda & Uganda | (2, 5) |
| Male provider role | Masculinities were threatened by women bringing home money or goods purchased with their own money, and men sometimes assumed this money or goods were from another man. | *These feelings of romantic jealousy heightened when a woman became financially independent or earned more than them* (4). | Rwanda & Uganda, Tanzania (men and women) | (2-4) |
| Hegemonic masculinities | Hegemonic masculinities were linked to how men were expected to behave in relationship (e.g. being hypersexual), and the power imbalance between men and women. | *Hence, the threat imposed by women’s empowerment on hegemonic masculinities helps us in understanding men’s negative attitude over women’s risen social and economic position and why it escalates jealousy in them and lead to violence perpetration against their female partners* (4). | Ecuador, Ethiopia, Rwanda & Uganda, Tanzania (men and women) | (1-5) |
| Femininities | Femininities were linked to how women were expected to behave in intimate relationships, and their ability to leave. | *Femininities were linked to being hyposexual (in contrast to men’s hypersexuality) and women were expected to be sexually satisfied by their husbands and not tempted by other men* (1). | Ecuador, Ethiopia, Tanzania (men and women) | (1, 3-5) |
|  |  |  |  |  |
| Upstream structural determinants linked to jealousy, infidelity and IPV | | | |  |
| Poverty | Poverty decreased men’s ability to have multiple partners, and led to women working outside of the home and becoming financially independent. | *Although new polygynous marriages were no longer common occurrences in the camp because of the economic hardship caused by displacement, a few participants described fearing the possibility of their husbands seeking other relationships or another wife and explained the ways in which they tried to prevent it* (5). | Ethiopia, Tanzania (men and women) | (3-5) |
| Displacement | Displacement decreased men’s ability to have multiple partners, and increased women’s ability to work outside of the home and express jealousy, which could result in conflict and IPV. | *[…] while women may have experienced romantic jealousy in Somalia […] in the refugee camp where polygyny is practiced less often, women were more empowered to challenge their husbands* (5). | Ethiopia | (5) |
| Protective factors against IPV | | | |  |
| Changing gender norms | Changing gender norms were characterized by women’s empowerment and decreased acceptance in men perpetrating violence, but could also cause male backlash and violence. | *Machismo was usually associated with older men, and there was a prevailing narrative that machistas were from a disappearing generation* (1). | Ecuador, Ethiopia, Tanzania (men) | (1, 4, 5) |
| Jealousy and infidelity perceived negatively | Negative views of jealousy and infidelity, usually that they are damaging to relationships and dangerous (including leading to IPV). | *Furthermore, jealousy was perceived as a negative trait which undermines patience and understanding among couples, and was cited as a common trigger of conflict and/or IPV […]* (2) | Ecuador, Ethiopia, Rwanda & Uganda, Tanzania (men and women) | (1-5) |
| IPV perceived negatively | IPV seen as a negative behaviour. | *Participants reported that physically forcing one’s wife to have sex was considered machista and socially unacceptable [...]* (1). | Ecuador | (1) |

**References**

1. Buller AM, Pichon M, Chevalier C, Treves-Kagan S. The role of gender and romantic jealousy in intimate partner violence against women, a mixed-methods study in Northern Ecuador. Cult Health Sex. 2022;25(2):223-40.

2. Kyegombe N, Stern E, Buller AM. "We saw that jealousy can also bring violence": A qualitative exploration of the intersections between jealousy, infidelity and intimate partner violence in Rwanda and Uganda. Soc Sci Med. 2022;292:114593.

3. Aloyce D, Mshana G, Peter E, Malibwa D, Buller AM, Mchome Z, et al. Pathways of romantic jealousy to intimate partner violence in Mwanza, northern Tanzania. Fam Relat. 2024;73(2):843-57.

4. Aloyce D, Stöckl H, Malibwa D, Peter E, McHome Z, Dwarumpudi A, et al. Men's reflections on romantic jealousy and intimate partner violence in Mwanza, Tanzania. VAW. 2023;29(6-7):1299-318.

5. Abudulai F, Pichon M, Buller AM, Scott J, Sharma V. Displacement, polygyny, romantic jealousy, and intimate partner violence: a qualitative study among Somali refugees in Ethiopia. Int J Environ Res Public Health. 2022;19(9):5757.
